# The “Belt and Road” Initiative and China's sporting goods exports: Basic characteristics and policy evaluation

**DOI:** 10.1016/j.heliyon.2024.e33189

**Published:** 2024-06-23

**Authors:** Rui Wang, Tingting Liang

**Affiliations:** a Business School, Guangzhou College of Technology and Business, Guangzhou, 510850, China; b School of Sports Media, Guangzhou Sport University, Guangzhou, 510500, China

**Keywords:** The “Belt and Road”, Sporting goods, Difference-in-differences, Policy evaluation

## Abstract

The paper analyzes the basic characteristics of China's sporting goods exports using data from the CEPII BACI database from 2007 to 2019, combined with the social network analysis method, and evaluates the policy effect of the “Belt and Road” initiative implementation on the impact of China's sporting goods exports using the difference-in-differences method. The research findings are as follows: 1) The overall scale of China's sporting goods exports has consistently expanded, marked by a rapid increase in the total export volume. The exported goods exhibit a comprehensive range of product categories, indicating an ongoing evolution in the structural composition of products. Geographically, the export destinations are widespread, covering a diverse range of countries. However, there is a noticeable concentration trend in sporting goods exports, with gymnastics and track and field equipment being the primary export commodities. These sporting goods predominantly penetrate markets in Southeast Asia, West Asia, and Eastern Europe. In the sporting goods trade network associated with the “Belt and Road” initiative, China holds a central role, with Thailand, Turkey, and Poland progressively advancing toward central positions. 2) The implementation of the “Belt and Road” initiative has had a positive impact on China's sporting goods exports, and the policy's influence is particularly significant on the countries and regions along the “Belt and Road”. The implementation of the policy does not favor the breathing growth of sporting goods exports, but it does promote the deepening of the export product. Based on these perspectives, it is imperative for China to establish a robust and sustainable trade network, proactively foster sporting diplomacy, maintain strategic focus, and enhance product quality to effectively propel the development of a sporting powerhouse.

## 1 Introduction

In 2013, Chinese President Xi Jinping first proposed a cooperative initiative to construct the “New Silk Road Economic Belt” and the “21st Century Maritime Silk Road” (the “Belt and Road” initiative). The “Belt and Road” initiative reflects China's unique solutions and wisdom in fostering a community with a shared future for humanity, while concurrently pursuing its own development. China has proactively fostered economic cooperation with countries and regions along the “Belt and Road”, ushering in a new era of bilateral relations. Within the framework of the “Belt and Road” cooperation, the sporting industry, as a crucial component of cultural exchanges, assumes a significant role in advancing the objectives of the “Belt and Road” initiative [1]. In 2017, President Xi presented a proposal to “accelerate the development of a sporting powerhouse” in the report of the 19th National Congress of the Communist Party of China. Subsequently, in 2019, the General Office of the State Council issued *the Notice of the Outline for the Construction of a Strong Sporting Country*, which explicitly emphasizes the need to expedite the establishment of a sporting powerhouse, reinforce the formulation of sporting policy planning, and fully leverage the pivotal role of sports in the new phase of building a comprehensively modern socialist country. In 2021, the State Sports General Administration of China issued the 14th *Five-Year Plan for Sports Development*, which provided a comprehensive roadmap for the advancement of the Chinese sporting industry and facilitated its high-quality growth. Consequently, it becomes particularly vital to promote the optimization of the export structure of sporting goods within China's sporting industry and enhance its significance and contribution to the national economy. Therefore, investigating whether the “Belt and Road” initiative contributes to the optimization of China's sporting goods exports structure is a question worthy of scholarly exploration.

The current scholarly discourse encompasses three primary categories of literature that delve into the determinants impacting China's sporting goods export. Firstly, political factors. The governance framework and behavioral norms within a nation are pivotal in ensuring the comprehensiveness of its national sports governance system and maintaining the coherence of its institutional structures and behaviors. This, in turn, fosters the sustainable development of the sports industry [2,3]. Institutions assume a critical role in the realm of foreign trade. In the context of bilateral trade, the direct institutional proximity between two nations can amplify the competitive dynamics, exerting a constraining effect on the advancement of bilateral trade [4]. The evolution of bilateral trade is contingent upon the openness of both participating entities. As the degree of openness among trading nations expands, there is a concomitant likelihood of an augmentation in the export volume of sporting goods [5]. Secondly, economic factors. A nation's GDP serves as a barometer of its economic magnitude. Put succinctly, an augmented GDP signifies heightened production capabilities for the exporting nation and amplified purchasing potential for the importing nation. An upswing in the aggregate GDP of the country of Destination proves advantageous in fostering an expanded appetite for sporting goods originating from the exporting country [6]. The genesis of trade transactions predominantly hinges on considerations of financial gain. Consequently, due regard must be accorded to the nuanced intricacies surrounding trade costs. Fixed trade costs, exemplified by geographical distances, assume an increasingly inhibitory role in bilateral trade dynamics as they escalate, thereby impeding export activities [7]. Simultaneously, escalating variable trade costs, such as labor expenditures, exert deleterious effects on the stability of sporting goods exports [8], thereby attenuating the competitive prowess of sporting goods within the export domain [9]. Investment, as one of the triumvirate propellers propelling economic growth, maintains an intricate nexus with exports. Foreign direct investment emerges as a catalyst in fostering the elevation of China's industrial framework, fortifying corporate competitiveness, consequently augmenting the trade volume associated with the export of sporting goods. Concurrently, amid the advancement of the information age, the pervasive accessibility of the Internet serves to alleviate the consumer lag resultant from information asymmetry, concurrently exerting a discernible impact on exports [5]. Furthermore, both enterprise heterogeneity [10] and consumer heterogeneity [5] wield considerable influence over the export dynamics of sporting goods from China. Thirdly, cultural factors. The shaping of a nation's sports culture is intricately linked to historical, geographical, and ethnic determinants. The costs and risks stemming from cultural disparities pose impediments to the elevation of China's sporting goods value chain [11]. Nevertheless, the escalating international resonance of China's sports culture progressively diminishes the inhibitory influence of cultural distance on the export of Chinese sports products [5].

The literature germane to this study can be classified into two primary categories, addressing the repercussions of the “Belt and Road” initiative on China's sporting goods trade. The first type of literature focuses on describing the status of sporting goods development and spatial pattern related to the “Belt and Road” initiative. With the “Belt and Road” initiative, China's sports industry has a new historic opportunity [12]. It is noteworthy that China's foreign trade in sporting goods has shown significant growth, and the overall trade volume continues to expand [13,14]. However, there are also bottlenecks, such as an inappropriate industrial structure, imperfect distribution and suppressed exports [15]. The overcapacity of the sporting goods sector and the undersupply of the sporting services sector have hampered the transformation of the sports industry from a secondary to a tertiary industry [16]. According to the global value chain theory, for the development of China's sporting goods industry, it is necessary to improve the driving mechanism, optimize the governance structure, promote industrial upgrading and achieve the transformation and upgrading of the industrial structure. These key measures are critical to promote high-quality development of China's sporting industry [17]. Furthermore, the “Belt and Road” initiative can break through the monopoly of Western economies, reduce trade costs and risks, and enhance the overall success rate of global value chain upgrading for China's sporting industry [11]. The “Belt and Road” initiative has emerged as a significant driver in accelerating the export of Chinese sporting goods enterprises, augmenting their international influence and competitiveness, and fostering innovation and research and development capabilities [18]. This initiative has fostered a close correlation between China and the trade space network of sporting goods with countries and regions along the “Belt and Road”. As a result, there has been an increase in trade activities between China and countries along the “Belt and Road”. These activities were facilitated by providing sporting facilities, developing infrastructure, economic upliftment, and cultural influence [19]. And, Cross-cultural sporting communication can promote the development of the sporting industry under the “Belt and Road” initiative [20]. However, despite this proximity, there remains substantial room for improvement in the sporting goods trade cooperation between them [21]. China's sporting manufacturing industry displays spatial agglomeration, similarly reflected in the sporting service industry's layout [22]. The second type of literature primarily focuses on examining the influencing factors of sporting goods trade in countries participating in the “Belt and Road” initiative, encompassing political, economic, and cultural aspects. Specifically, it is observed that the larger the market scale, the stronger the political stability, and the higher the degree of openness in countries and regions along the “Belt and Road” initiative, the greater the volume of sporting goods exported from China [23]. On the contrary, factors such as market scale, political stability, degree of openness, cultural distance, and geographical distance between countries can impede sporting goods exports within the “Belt and Road” initiative [6]. Moreover, entrepreneurial capital also plays a significant role in influencing the export of sporting goods [24]. Hence, extant literature predominantly centers on scrutinizing the influence of political factors, economic factors, and cultural factors on China's exportation of sporting goods. Furthermore, it delves into the transformative developments within the Chinese sports industry and sporting goods sector within the context of the “Belt and Road” initiative. This body of work offers invaluable guidance and insights for stakeholders involved in the decision-making processes pertaining to the export trade of Chinese sporting goods. The principal objective of this study is to scrutinize, through the lens of policy effectiveness, the ramifications of the introduction of the “Belt and Road” initiative on the export dynamics of sporting goods from China.

Research findings can be summarized as follows. The aggregate volume of China's sporting goods exports manifests a persistent expansion. However, this growth trajectory is subject to fluctuations attributed to factors such as the financial crisis, the “new normal” of China's economy, and trade frictions. Furthermore, a discernible trend is observed wherein the total exports of sporting goods from China to “Belt and Road” countries and regions exhibit a consistent upward trajectory in contrast to non- Belt and Road countries and regions. The exported sporting goods encompass a diverse array of product categories, yet the product structure reveals an imbalance, marked by a pronounced inclination towards concentration. The primary focal points within sporting goods exports include sports footwear, gymnastics and track and field equipment, as well as ball sports equipment and supplies. Conversely, the developmental progression of rifles and other sports shooting equipment and sportswear and protective gear remains comparatively inadequate. Geographically, sporting goods exports display a broad distribution, mainly exported to Southeast Asia, West Asia, and Eastern Europe. Within the framework of the “Belt and Road” sporting goods trade network, China occupies a relatively central position, with countries such as Thailand, Turkey, and Poland gradually gravitating towards central roles over time. Empirical outcomes underscore a noteworthy positive influence stemming from the implementation of the “Belt and Road” initiative on China's sporting goods exports. This impact is particularly pronounced when compared with non-Belt and Road countries and regions, with “Belt and Road” countries and regions experiencing a more substantial influence. Through a meticulous examination of export triple margins, it is discerned that policy implementation impedes the expansion in breadth of sporting goods exports. Conversely, it fosters an augmentation in the depth of exported products.

This paper contributes to the existing literature in several aspects. Firstly, in contrast to prior studies primarily focusing on the developmental aspects of China's sporting industry or sporting goods within the framework of the “Belt and Road” initiative [11,12,15,[18], [19], [20]], this paper employs the “Belt and Road” Initiative as a quasi-natural experiment to scrutinize the policy effects on China's sporting goods exports. This not only broadens the research scope on the “Belt and Road” initiative and sporting goods exports but also unveils a more specific mechanism through which the initiative influences the export dynamics of sporting goods. Secondly, departing from the prevalent focus in the literature on bilateral relationships between China and the sporting industry of individual countries [19,20], this study combines social network analysis with traditional mathematical statistical methods. The goal is to investigate the positioning of China and countries and regions along the “Belt and Road” in the “Belt and Road” sporting goods trade network. This methodological integration provides a holistic comprehension of the relationships and positions of countries and regions in the sporting goods trade, thereby contributing to the literature by offering a nuanced analysis of the characteristics of sporting goods exports. Furthermore, it expands the research boundaries of sporting goods export within the overarching framework of the “Belt and Road” initiative. Finally, situated at a crucial juncture where China actively aspires to the status of a sporting power and integrates the sporting industry into the national economy as a pivotal sector, a comprehensive and systematic review of the impact of the “Belt and Road” initiative on China's sporting goods exports becomes paramount. This not only addresses existing gaps in the literature concerning the effects of the “Belt and Road” initiative but also provides a practical foundation for China to strategically enhance the international influence of its sporting culture through the “Belt and Road” framework.

The structure of this paper is outlined as follows. The second section will offer a detailed description of the materials and methods utilized in this paper. Subsequently, the third part will present an overview of the current characteristics of China's sporting goods exports. In the fourth section, a comprehensive analysis and evaluation of the policy effects of the “Belt and Road” initiative on China's sporting goods exports will be presented. Finally, the concluding remarks will be provided in the last section.

## 2 Methods and materials

### 2.1 Methods

#### 2.1.1 Social network analysis

Social network analysis is a research methodology in sociology. According to sociological theory, society is not formed solely by isolated individuals, but rather by interconnected networks comprising of nodes and the relationships between them. Social network analysis aims to explore the structure and attributes of these networks through the analysis of relationships within them. In this paper, social network analysis is employed to examine the positions of countries and regions along the “Belt and Road” initiative in the sporting goods trade network. Social network analysis serves as a valuable tool for expeditiously discerning distribution patterns and competitive dynamics inherent in the network, thereby facilitating a nuanced understanding of China's specific positioning and relational dynamics within the “Belt and Road” sporting goods trade network. Analyzing the structure of the trade network provides insights into the roles played by different countries in the sporting goods trade network and their interdependence. Such insights are of paramount significance in steering the development of judicious trade policies and fostering collaborative agreements. Following the approach of Li et al. [25] and Qi et al. [26], this paper employs social network analysis to investigate the positions of countries and regions along the “Belt and Road” in the sporting goods trade network. In this context, each node in the network represents an economy, and the size of the node is determined by its degree of centrality within the “Belt and Road” sporting goods trade network. A larger node indicates a more significant position of the economy within the trade network. Furthermore, the thickness of the edges represents the level of sporting product trade between economies, with thicker edges indicating higher levels of trade between them.

#### 2.1.2 DID method

The DID method is a widely used research technique in the field of econometrics. It is primarily employed for evaluating the effects or impacts of specific policies, intervention measures, or treatments on individuals, organizations, or regions. The fundamental concept behind the DID method involves estimating the effects of a policy or intervention by comparing the differences between a treatment group and a control group before and after the intervention. The essential aspect of this approach is to separate differences in pre- and post-intervention periods between the treatment and control groups from the effects of time and individual-specific characteristics, allowing for causal inference.

This paper adopts from Chen et al. [27], Chen and Zhou [28], and Duflo [29], who exploited China's send-down movement, the famine in China, and the INPRES program as a quasi-natural experiment, respectively. Similarly, the “Belt and Road” initiative is viewed as a quasi-natural experiment, and the DID method is employed in this empirical study to examine its impact on China's sporting goods exports. The DID method is selected for several reasons. Firstly, compared to other empirical research methods, the DID method can mitigate the impact of selection bias by comparing the differences between the treatment and control groups before and after the intervention. The growth of the control group is considered natural growth, while the growth of the treatment group combines natural growth and the impact of the intervention. By comparing before the intervention, the DID method can control some potential unobservable factors and provide a more accurate estimate of the intervention's effect. Secondly, the DID method can address time trends, wherein the dependent variable may change over time before and after policy implementation. The DID method can eliminate the impact of this time trend by comparing changes in the treatment and control groups, providing more precise estimation results. This helps to eliminate other factors that may cause changes, making it easier to attribute them to policy interventions. Finally, the DID method can better resolve the causal effect problem compared to other research methods by comparing the differences between the treatment and control groups before and after policy implementation. This is because the DID method can control the effects of individual characteristics that remain constant over time and unobserved individual characteristics. By comparing the differences between the treatment and control groups, the impact of policy on sports goods exports can be more reliably inferred. It should be noted that the DID method has some limitations. The DID method theoretically requires that the treatment and control groups have similar trends before the intervention. If this assumption holds, then the DID method can estimate the policy effect more accurately while eliminating trend changes caused by other factors. Therefore, this article conducts a parallel trend test in the empirical section. In summary, it is reasonable for this paper to utilize the DID method to analyze the policy effect of the “Belt and Road” initiative on the export of Chinese sports goods.

### 2.2 Materials

#### 2.2.1 Sample selection and data sources

The “Belt and Road” initiative traces its origins back to the historical Silk Road, a network of ancient land-based commercial trade routes that interconnected Asia, Africa, and Europe. The Silk Road was instrumental in fostering trade, cultural exchange, and diplomacy between the regions it linked. It consisted of two major sections: the Overland Silk Road and the Maritime Silk Road, which now serve as the precursors to what we currently refer to as the “New Silk Road Economic Belt” and the “21st Century Maritime Silk Road.” Initially established to facilitate the transportation of valuable commodities like silk and other Chinese-made goods, the Silk Road gradually transformed into a vital conduit for broader political, economic, and cultural exchanges between the Eastern and Western civilizations. This historical trade route played a pivotal role in disseminating knowledge, ideas, and technologies across different regions, contributing to the enrichment and mutual understanding of diverse cultures.

During a visit to Central Asia and Southeast Asia in 2013, President Xi Jinping proposed the “New Silk Road Economic Belt” and the “21st Century Maritime Silk Road,” which garnered significant international attention. To facilitate the implementation of the “Belt and Road” initiative, the Chinese government issued the *Vision and Actions on Jointly Building Silk Road Economic Belt and* 21st*-Century Maritime Silk Road* in March 2015. The “Belt and Road” initiative embodies the principles of co-business, co-build, and co-share, along with the core concepts of peace and cooperation, openness and tolerance, learning, and mutual benefit. According to data from the “Belt and Road” Network, as of November 23, 2021, China has engaged in the 207 “Belt and Road” cooperation documents with 141 countries and 32 international organizations. The construction of the “Belt and Road” has yielded remarkable achievements, while the international consensus on jointly building the initiative continues to expand.

This study proposes employing the “Belt and Road” initiative as a quasi-natural experiment to assess its impact on the export of sporting goods. The treatment group comprises countries and regions involved in the “Belt and Road” initiative, while the non-Belt and Road countries and regions constitute the control group. The application of the DID method is employed to calculate and compare the effects of the “Belt and Road” initiative on the export of sporting goods between these two groups. This methodological approach is instrumental in isolating the impact of the “Belt and Road” initiative on sporting goods exports by controlling for any pre-existing differences between the treatment and control groups. Through the utilization of this method, the paper aims to gain a more comprehensive understanding of the effectiveness of the “Belt and Road” initiative in promoting the export of sporting goods from China.

Furthermore, the following preprocessing steps were undertaken during the sample selection process for this paper:

(1) To comprehensively analyze the development characteristics of China's sporting goods exports to countries and regions along the “Belt and Road”, this paper employs various relevant international and national classification standards. The utilization of internationally recognized classification standards enhances the comparability and reliability of the findings, contributing to a more meaningful understanding of the dynamics and trends in the trade of sporting goods between China and the “Belt and Road” countries and regions. The international classification standards used include the UN Comtrade database (HS2007) and CEPII BACI (HS2007). Additionally, some national classification standards, namely the Classification of National Economic Industries (GB/T 4754-2017) and the Statistical Classification of the Sporting Industry (2019), are consulted. Moreover, the classification of sporting goods by Ji and Gu is also referenced [30]. Based on these sources, the paper categorizes sporting goods into ten distinct categories, which are as follows: sportswear and protective gear, sports footwear, sports fields and related equipment, water sports equipment and supplies, ice and snow sports equipment and supplies, ball sports equipment and supplies, fishing gear equipment, gymnastics and track and field equipment, rifles and other sporting equipment and other sporting goods and equipment. The classification rules detailing the assignment of sporting goods to these categories are presented in Table 1.

**Table 1:** Detailed rules for classification of sporting goods.

Category	Product code	Product subclass code	Product category
1	HS4203	HS420321	Sportswear and protective gear
2	HS6402	HS640219	Sports footwear
	HS6403	HS640319	
	HS6404	HS640411	
3	HS8432	HS843280、HS843290	Sports fields and related equipment
	HS8433	HS843311、HS843319	
4	HS8903	HS890391、HS890399、HS890310、HS890392	Water sports equipment and supplies
	HS9506	HS950621、HS950629	
5	HS6402	HS640212	Ice and snow sports equipment and supplies
	HS6403	HS640312	
	HS9506	HS950611、HS950612、HS950619、HS950670	
6	HS9506	HS950631、HS950632、HS950639、HS950640、HS950651、HS950659、HS950661、HS950662、HS950669	Ball sports equipment and supplies
	HS9504	HS950420	
7	HS9507	HS950710、HS950720、HS950730、HS950790	Fishing gear equipment
8	HS9506	HS950691	Gymnastics and track and field equipment
9	HS9303	HS930320、HS930330	Rifles and other sports shooting equipment
10	HS9504	HS950410、HS950430、HS950440、HS950490	Other sporting goods and equipment
	HS9506	HS950699
(2) The CEPII database, encompassing data from over 200 countries and regions worldwide, serves as a comprehensive source of bilateral trade data for each nation. Consequently, this paper initiates the process by consolidating global bilateral trade data derived from the CEPII BACI database, which meticulously records bilateral trade transactions for individual countries. Subsequently, this paper further refines the dataset to focus specifically on bilateral trade data for sporting goods, utilizing the aforementioned classification for such products. In the refinement process, adhering to the principle of maximizing the inclusion of countries with available data, the paper establishes a research sample consisting of balanced panel data. This dataset specifically pertains to China's sporting goods exports to a total of 177 countries and regions. For a comprehensive listing, please refer to Appendix 1.
(3) The treatment group selected for this paper comprises the countries along the “Belt and Road” initiative as delineated by China's “Belt and Road” network, rather than encompassing all countries and regions that have entered into cooperative agreements pertaining to the “Belt and Road” initiative with China. As per the data sourced from China's “Belt and Road” Network, a total of 65 countries and regions align with the “Belt and Road” initiative. Excluding China, there are 64 countries and regions. However, owing to substantial data gaps in various metrics pertaining to Bhutan, data availability considerations dictated the exclusion of Bhutan from the final sample. Consequently, the ultimate sample consists of 63 countries along the “Belt and Road” initiative, factoring in the aforementioned criteria and adjustments. To conduct a comprehensive analysis of the regional distribution of China's sporting goods exports, this paper utilizes data from the China's “Belt and Road” Network and classifies the 63 countries and regions along the “Belt and Road” initiative based on their intercontinental distribution. The distribution data is presented in Table 2.

**Table 2:** Intercontinental distribution of the “Belt and Road” countries and regions.

The continent	country
Africa (1)	Egypt (EGY)
Europe (20)	Albania(ALB)、Belarus(BLR)、Bosnia & Herzegovina(BIH)、Bulgaria(BGR)、Croatia(HRV)、Czech Republic(CZE)、Estonia(EST)、Hungary(HUN)、Latvia(LVA)、Lithuania(LTU)、Moldova(MDA)、Montenegro(MNE)、Poland(POL)、Republic of Macedonia(MKD)、Romania(ROU)、Russian Federation(RUS)、Serbia(SRB)、Slovakia(SVK)、Slovenia(SVN)、Ukraine(UKR)
Asia (42)	Afghanistan(AFG)、Armenia(ARM)、Azerbaijan(AZE)、Bahrain(BHR)、Bangladesh(BGD)、Brunei(BRN)、Cambodia(KHM)、Georgia(GEO)、India(IND)、Indonesia(IDN)、Iran(IRN)、Iraq(IRQ)、Israel(ISR)、Jordan(JOR)、Kazakhstan(KAZ)、Kuwait(KWT)、Kyrgyzstan(KGZ)、Laos(LAO)、Lebanon(LBN)、Malaysia(MYS)、Maldives(MDV)、Mongolia(MNG)、Myanmar (MMR)、Nepal(NPL)、Oman(OMN)、Pakistan(PAK)、Palestinian territories(PSE)、Qatar(QAT)、Saudi Arabia(SAU)、Singapore(SGP)、Sri Lanka(LKA)、Syria(SYR)、Tajikistan(TJK)、Thailand(THA)、The Philippines(PHL)、Timor Leste (TLS)、Turkey(TUR)、Turkmenistan(TKM)、United Arab Emirates(ARE)、Uzbekistan(UZB)、Vietnam(VNM)、Yemen(YEM)
(4) To mitigate the influence of external factors, notably the COVID-19 pandemic, this paper has designated the research period spanning from 2007 to 2019. The impact of the ongoing China-U.S. trade war has not been considered in this study for the following reasons. Firstly, the sample data used in this study covers the period from 2007 to 2019, while the China-U.S. trade war occurred from 2018 to 2022. Therefore, it is difficult to capture the impact of the trade war on the data for only two years, and it should be noted that the impact of the trade war on the data may have a lag effect. Secondly, this study primarily focuses on the Belt and Road Initiative, dividing countries and regions into those along the Belt and Road and those not along the Belt and Road. It is difficult to define the impact of the trade war on the data and to distinguish which countries were affected by the trade war. Lastly, the China-U.S. trade war targeted China directly, particularly in industries such as aviation, information and communication technology, robotics, pharmaceuticals, and machinery, and had a relatively limited impact on Chinese sports goods exports [31]. We will further explore the impact of the China-U.S. trade war in this context in the future. The variables utilized in this manuscript are sourced as follows: firstly, data concerning the export value of sporting goods and the triple margins for sporting goods are extracted from the CEPII BACI database. Secondly, control variables are sourced from a range of reputable databases, including the World Bank, CEPII, annual reports from the *Wall Street Journal and the Heritage Foundation*, as well as data from the WTO official website and international sports organizations. As a result of these meticulous data gathering processes, the paper culminates in the compilation of a balanced panel dataset spanning 177 countries from the years 2007–2019. Leveraging this dataset, the paper endeavors to furnish a dependable and precise evaluation of the impact of the “Belt and Road” initiative on China's sporting goods exports.

#### 2.2.2 Model settings

To evaluate the policy effects of the “Belt and Road” initiative on China's sporting goods exports, this paper employs the DID method to empirically test the impact of the initiative. As the “Belt and Road” initiative was proposed towards the end of 2013 and officially included in the government work report in March 2014, this paper adopts the approach taken by Sun and Qin [32] and treats the implementation of the “Belt and Road” initiative as a quasi-natural experiment, designating the year 2014 as the time when the policy shock event occurred. In this paper, the treatment group consists of the 63 countries and regions participating in the “Belt and Road” initiative, while the remaining countries and regions serve as the corresponding control group (Table 2). The basic estimation model is formulated as follows:

(1) $$Y_{it}=\alpha_{0}+\alpha_{1}post_{it}+\alpha_{2}treat_{it}+\alpha_{3}post_{it}\times treat_{it}+\gamma X+\mu_{i}+\nu_{t}+group_{g}\times year_{t}+\varepsilon_{it}$$

where *i* represents a country, *t* represents a year. *Y*_*it*_ corresponds to the export value of sporting goods exporting to country *i* at time *t*, and the paper incorporates the decomposition of sporting goods exports into extensive margin (*EM*) and intensive margin (*IM*) (including price margin (*P*) and quantity margin (*Q*)) into the model in order to examine both the breadth and depth of exports. The dummy variable *post*_*it*_ indicates before and after the implementation of the “Belt and Road” initiative. The dummy variable *treat*_*it*_ is a grouping of countries. The dummy variable *post*_*it*_ × *treat*_*it*_ is the interaction term between country grouping and policy implementation and is the core explanatory variable in this paper. X represents other control variables, *μ*_*i*_ denotes factors that do not vary with country *i*, *ν*_t_ denotes factors that do not vary over time *t*. To further control for the characteristics of sporting goods across years, this paper introduces an interaction item *group*_*g*_ × *year*_*t*_ for the sporting goods category and year dummy variables in the model to control for factors that change over time at the sporting goods category level, where *g* represents product category. *ε*_it_ is a random perturbation term. The estimated coefficient α_3_ is the focus of this paper, which indicates the effect of the implementation of the “Belt and Road” initiative. If *α*_*3*_ > 0, it indicates that the implementation of the initiative has boosted trade in sporting goods, while α_3_ < 0 indicates that the implementation of the initiative has inhibited the growth of trade in sporting goods.

#### 2.2.3 Variable descriptions

##### 2.2.3.1 Interpreted variables

Export value of sporting goods (ln*export*): it is represented by the natural logarithm of China's sporting goods exports to 177 countries and regions worldwide from 2007 to 2019 in the CEPII BACI database.

Under the aforementioned classification of sporting goods, this paper utilizes the sporting goods export data from the CEPII BACI database. Additionally, drawing insights from previous studies conducted by Hummels and Klenow [33], Qian [34] and Shi [35], the present paper decomposes sporting goods exports into dual margin: the extensive margin (EM) and the intensive margin (IM). Furthermore, the intensive margin is further disaggregated into the price margin (P) and the quantity margin (Q), thus forming a ternary margin for sporting goods. This analytical approach is employed to discern the sources of the impact of the “Belt and Road” initiative on China's sporting goods exports.

First, this paper divides the growth of China's sporting goods exports into the extensive margin and the intensive margin. The function is as follows:

(2) $$EM_{eijt}=\Sigma_{j\in I_{eijt}}p_{wijt}q_{wijt}/\Sigma_{j\in I_{wijt}}p_{wijt}q_{wijt}$$

(3) $$IM_{eijt}=\Sigma_{j\in I_{eijt}}p_{eijt}q_{eijt}/\Sigma_{j\in I_{eijt}}p_{wijt}q_{wijt}$$

where *e* represents the exporting country, *i* represents the destination country, *w* represents the reference country (the world), *j* represents the product (referring to sporting goods in this article), and *t* represents the time.

*p*_*eijt*_ and *p*_*wijt*_ respectively represent the prices of exports of product *j* from country *e* and country *w* to country *i* at time *t*, and *q*_*eijt*_ and *q*_*wijt*_ represent the quantities of products *j* exported by country *e* and country *w* to country *i* at time *t*. *I*_*eijt*_ denotes the set of products *j* exported by country *e* to country *i* at time *t*, while *I*_*wijt*_ denotes the set of products *j* exported by country *w* to country *i* at time *t*. *EM*_*eijt*_ indicates the extensive margin of products *j* exported by country *e* to country *i* at time *t*, referring to the share of world exports that overlap with the portion of exports from country *e* to country *i* in total world exports, reflecting the breadth of export of country *e*. *IM*_*eijt*_ denotes the intensive margin of products *j* exported by country *i* to country *i* at time *t*, referring to the share of total exports from country *e* in the portion of world exports that overlap with exports from country *e* to country *i*, reflecting the depth of the products exported from country *e*.

Second, this paper decomposes the intensive margin into price margin and quantity margin, The function is as follows:

(4) $$IM_{eijt}=P_{eijt}\times Q_{eijt}$$

(5) $$P_{eijt}=\Pi_{j\in I_{eijt}}{\left[p_{eijt}/p_{wijt}\right]}^{w_{eij}}$$

(6) $$Q_{eijt}=\Pi_{j\in I_{eijt}}{\left[q_{eijt}/q_{wijt}\right]}^{w_{eij}}$$

where *P*_*eijt*_ is the price margin, representing the quality of sporting goods, and Q_*eijt*_ represents the quantity margin. *w*_*eij*_ denotes the weights. The function is as follows:

(7) $$w_{eij}=\frac{\left(h_{eijt}-h_{wijt}\right)/\left(\ln\;h_{eijt}-\ln\;h_{wijt}\right)}{\Sigma_{j\in I_{eijt}}\left[\left(h_{eijt}-h_{wijt}\right)/\left(\ln\;h_{eijt}-\ln\;h_{wijt}\right)\right]}$$

(8) $$h_{eijt}=p_{eijt}q_{eijt}/\Sigma_{j\in I_{eijt}}p_{eijt}q_{eijt}$$

(9) $$h_{wijt}=p_{wijt}q_{wijt}/\Sigma_{j\in I_{eijt}}p_{wijt}q_{wijt}$$

Here, *h*_*eijt*_ indicates the proportion of country *e* exporting product *j* to country *i* in the period *t*, *h*_*wijt*_ denotes the share of the overlap between exports from country *w* and *e* to country *i* for product *j*.

##### 2.2.3.2 Core explanatory variables

The core explanatory variable in this paper is *post*_*it*_ × *treat*_*it*_, which represents the interaction between country grouping and policy implementation. It is a measure of whether a policy is effective. The dummy variable *post*_*it*_ indicates before and after the implementation of the “Belt and Road” initiative, with *post*_*it*_ = 1 being after the “Belt and Road” initiative (2014–2019) and *post*_*it*_ = 0 being before the initiative (2007–2013). The dummy variable *treat*_*it*_ is a grouping of countries, with *treat*_*it*_ = 1 representing the “Belt and Road” countries and regions (treatment group) and *treat*_*it*_ = 0 representing non-Belt and Road countries and regions (control group).

##### 2.2.3.3 Control variables

The level of economic development (ln*pgdp*): it is represented by the natural logarithm of per capita GDP, which is derived from data available in the World Bank database. This metric serves as a fundamental measure of a country's economic performance, effectively reflecting the level of economic development and the degree of advancement within a nation. It offers valuable insights into a country's economic strength, market size, as well as income level, and consumption capacity of its population.

Official exchange rate (ln*oer*): it is represented as the natural logarithm of the official exchange rate sourced from the World Bank database. Official exchange rate is calculated as an annual average based on monthly averages (local currency units relative to the U.S. dollar). Fluctuations in the exchange rate can exert noteworthy effects on the economic growth, employment, and consumption patterns of both domestic and foreign economies. Furthermore, these exchange rate fluctuations can also influence the pricing, output, and diversity of products exported by firms [36].

Openness (*openness*): In this paper, data on “Trade Freedom” and “Investment Freedom” are sourced from the annual report published by the *Wall Street Journal and the Heritage Foundation* of the United States. Building on the measure of *openness* proposed by Li and Zhai [37], the index of a country's openness to the outside world is computed by applying a 50 % weighting to “Trade Freedom” and “Investment Freedom.” A higher index value indicates a greater degree of openness, implying a more conducive environment for establishing trade relations.

Population density (ln*pop*): It is represented by the natural logarithm of the number of people per kilometer of land area, obtained from the World Bank database. This variable reflects the size of a country's market, and a higher population density is associated with potential benefits in terms of reduced transportation, information, and marketing costs, thereby facilitating trade generation.

Geographical distance (ln*dis*): It is measured as the distance between the two capitals in the CEPII database. The geographical distance between China and other countries or regions is expected to influence transportation, transaction, and other trade-related costs, thus affecting the overall foreign trade activities between the countries. Moreover, differences in geographical locations may lead to variations in the utilization of natural resources and trade patterns, making geographical distance a significant factor impacting foreign trade.

Whether or not to join *the WTO* (*wto*): This binary variable indicates whether the destination country is a member of the World Trade Organization (WTO). A value of 1 denotes membership, while 0 represents non-membership. WTO membership status can have implications for trade relations and cooperation between countries, potentially affecting trade facilitation and regulatory harmonization.

Whether or not to hold an international sporting event (*sport*): This binary variable indicates whether the destination country has hosted an international sporting event. A value of 1 indicates that an international sporting event has been held in the country, while a value of 0 indicates no such event. Hosting international sporting events can influence a country's exposure, visibility, and reputation on the global sporting stage, which may, in turn, impact its sporting trade relations and opportunities.

##### 2.2.3.4 Descriptive statistical

Based on the principle of ensuring data availability, the final study sample consists of balanced panel data on China's sporting goods exports to 177 countries and regions. Descriptive statistics of the variables are presented in Table 3, while the results of a simple difference test are reported in Table 4. As observed in Table 3, the standard deviation of China's sporting goods exports is 2.646, while the average value is 9.112. This indicates substantial variations in China's sporting goods exports to different countries. It is noteworthy that the data on the price margin and quantity margin reveal that changes in the intensive margin are mainly caused by fluctuations in the price margin. Turning to the results in Table 4, they indicate that following the proposal of the “Belt and Road” initiative, China's sporting goods exports to countries and regions along the “Belt and Road” have experienced significant growth in both total exports and their breadth and depth. These findings demonstrate that the “Belt and Road” initiative plays a vital role in promoting the expansion of China's sporting goods exports, with a more pronounced effect evident in the “Belt and Road” countries and regions. These results underscore the positive impact of the “Belt and Road” initiative on China's trade in sporting goods, indicating the initiative's potential in fostering economic development and trade cooperation with partner countries and regions along its routes. Moreover, the observed growth in sporting goods exports reflects the initiative's role in facilitating enhanced connectivity and economic collaboration between China and the participating countries and regions.

**Table 3:** Descriptive statistical.

Variable name	Variable meaning	Observations	Mean	SD	Min	Max
ln*export*	Export value of sporting goods	1730	9.112	2.646	0.449	16.432
*EM*	Sporting goods of extensive margin	1730	0.836	0.229	0.010	1
*IM*	Sporting goods of intensive margins	1730	0.345	0.198	0.001	0.961
*P*	Sporting goods of quantity margins	1730	0.346	0.199	0.001	0.961
*Q*	Sporting goods of marginal	1730	0.994	0.004	0.976	1.000
ln*pgdp*	Level of economic development	1730	8.483	1.340	5.749	11.390
ln*oer*	Official exchange rate	1730	3.555	2.479	0.194	10.645
*openness*	Openness	1730	64.074	13.345	29.800	92.500
ln*pop*	Population density	1730	4.209	1.444	0.982	8.993
ln*dis*	Geographical distance	1730	9.029	0.545	7.063	9.858
*wto*	Whether or not to join the WTO	1730	0.918	0.275	0	1
*sport*	Whether or not to hold an international sporting event	1730	0.166	0.373	0	1

Note: The above descriptive statistics are based on non-missing values of all variables.

**Table 4:** Differential tests.

Variable name	Control group			Treatment group			DID
	Before the policy	After the policy	difference	Before the policy	After the policy	difference	
ln*export*	8.684	9.094	0.410***	9.253	9.879	0.626***	0.147**
*EM*	0.757	0.844	0.087***	0.853	0.914	0.061***	−0.034**
*IM*	0.303	0.316	0.013	0.341	0.361	0.020	0.018
*P*	0.305	0.318	0.013	0.342	0.362	0.020	0.018
*Q*	0.993	0.993	0.000	0.994	0.994	0.001**	0.001**

Note: ***, **, * indicate statistical significance at the 1 %, 5 %, and 10 % levels respectively.

## 3 The “Belt and Road” initiative and China's sporting goods exports: basic features

### 3.1 General characteristics

**Fig. 1:**
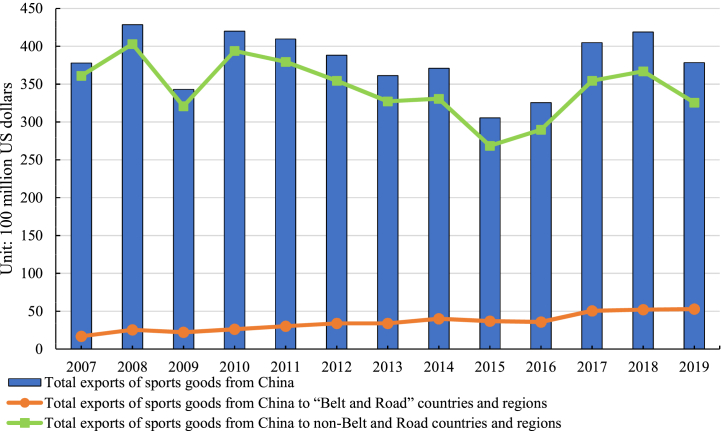
The total exports of sporting goods from China to global, the “Belt and Road”, and non-Belt and Road countries and regions.

With the rapid development of the Chinese economy, the sporting industry's position within the national economy has become increasingly significant, leading to substantial growth in the foreign trade of sporting goods. Analysis of data from the CEPII BACI database, encompassing China's exports of sporting goods to 177 countries and regions globally, reveals a consistent upward trajectory in total export value since 2007. The total export value increased from $37.793 billion in 2007 to $37.855 billion in 2020 (Fig. 1). Notably, two noticeable declines occurred in 2009 and 2015, with growth rates of −19.94 % and −17.68 %, respectively. These declines can be attributed to the considerable impact of the global financial crisis in 2008, which severely disrupted the international trade market and led to a sharp decline in China's sporting goods exports in 2009, resulting in clear negative growth. Furthermore, beginning in 2012, China's GDP growth rate witnessed a slowdown, signifying a shift in the stage of economic growth and reflecting the emergence of a “new normal” in the Chinese economy. Under the “new normal” of the economy, several factors have contributed to China's economic growth slowdown, including insufficient domestic demand and consumption capacity, a declining labor supply, the waning demographic dividends, and a deceleration in total factor productivity growth. These factors played a significant role in the decrease in sporting goods exports from 2015 to 2016. Additionally, China has encountered a rising number of trade frictions over the past decade, characterized by the proliferation of external trade barriers [13]. As evidenced by data released by the Trade Remedy Investigation Bureau of the Chinese Ministry of Commerce, China received a total of 215 trade remedy complaints between 2015 and 2016, accounting for approximately one-third of the total number of trade remedy cases filed globally. These trade frictions have also been an important contributing factor to the decline in sporting goods exports during the aforementioned period. The trend in China's exports of sporting goods to non- “Belt and Road” countries and regions has generally mirrored the overall trend, declining from $36.097 billion in 2007 to $32.554 billion in 2019, representing an average of 91.05 % of the total export value each year. However, most countries and regions along the “Belt and Road” are developing nations with relatively lower levels of economic development, potentially rendering them less susceptible to international economic fluctuations compared to non- “Belt and Road” countries and regions. Consequently, China's exports of sporting goods to the “Belt and Road” countries and regions have demonstrated a steady upward trajectory with smaller fluctuations. In 2019, the export value reached $5.3 billion, more than three times the value in 2007. This consistent upward trend suggests significant improvement in the development of the sporting industry, despite fluctuations in the overall growth rate of China's sporting goods exports.

### 3.2 Export product structure

**Fig. 2:**
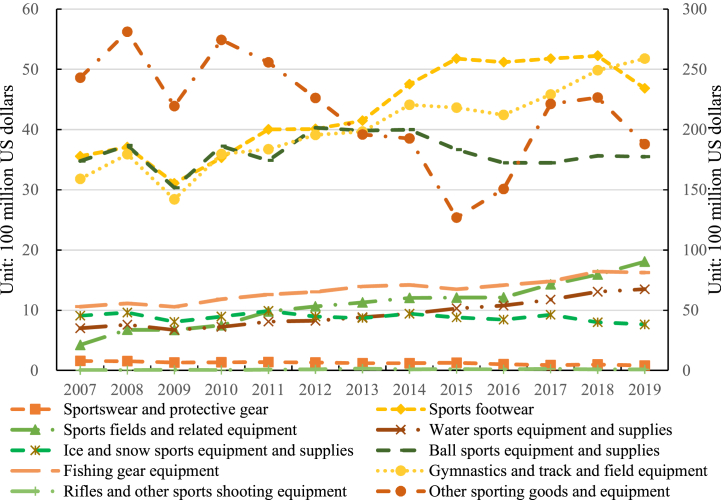
Total exports of 10 categories of sporting goods from China to the world. *Notes:* The left axis indicates the export value of the first nine types of sporting goods, and the right axis shows the exports value of other sporting goods and equipment. The same as below.

**Fig. 3:**
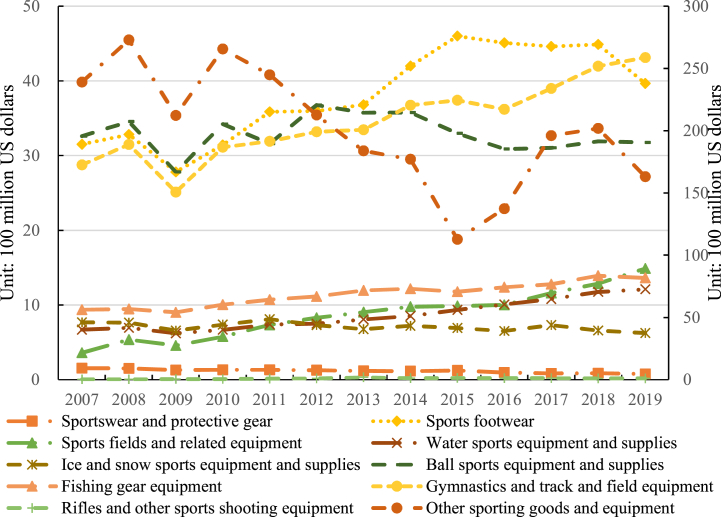
Total exports of 10 categories of sporting goods from China to non- Belt and Road countries and regions.

**Fig. 4:**
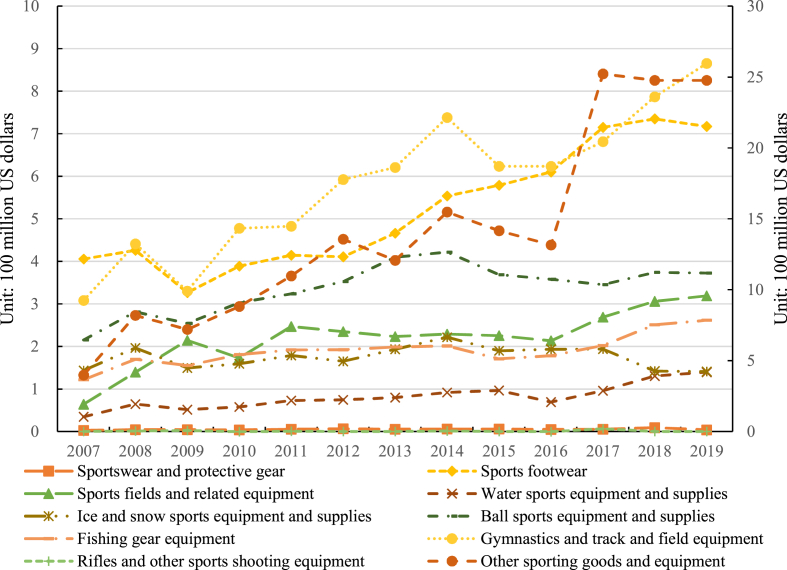
Total exports of 10 categories of sporting goods from China to the “Belt and Road” countries and regions.

The total exports of ten categories of sporting goods from China to the global, the “Belt and Road” countries and regions, and non-Belt and Road countries and regions are presented in Fig. 2, Fig. 3, and Fig. 4, respectively. Fig. 2 illustrates the fluctuating upward trend in Chinese sporting goods exports from 2007 to 2019. Specifically, the results show that ball sports equipment and supplies experienced the highest export volume and exhibited the most significant fluctuations. The export value for ball sports equipment and supplies increased from $3.479 billion in 2007 to $3.549 billion in 2019, with a cumulative export value of $47.164 billion. The average annual export value was $3.628 billion, with an average annual growth rate of 0.17 %. This highlights that China's sporting goods exports are primarily concentrated in ball sports equipment and supplies, as well as gymnastics and track and field equipment, with the latter category maintaining an average annual level of around 11 % of the total (Fig. 2).

China's exports of sporting goods were predominantly concentrated in sports footwear, gymnastics and track and field equipment, and ball sports equipment and supplies, while the development level in rifles and other sports shooting equipment and sportswear and protective gear was comparatively insufficient. The genesis of this structure can be attributed to the advanced state of development in the Chinese manufacturing industry. Concurrently, Chinese sports enterprises have strategically positioned themselves in the global market by actively engaging in or hosting international sports events, coupled with the formulation of judicious market positioning strategies [38,39]. This multi-layered driving force has endowed Chinese sporting goods with formidable competitiveness on the global stage, securing a broader international market share for the nation's sports industry.

Furthermore, the trends in China's exports of various sporting goods to non-Belt and Road countries and regions generally align with the overall trend. The main sporting goods exported by China to these countries include sports footwear, gymnastics and track and field equipment, and ball sports equipment and supplies in Fig. 3. In 2015, China witnessed a significant quantity of sports footwear exported to non-Belt and Road countries and regions, amounting to an export value of $4.601 billion. The second-highest category was gymnastics or competitive equipment and supplies, with a cumulative export value of $5.238 billion. Notably, the highest export value was recorded in 2019, reaching $4.314 billion. Sports fields and related equipment experienced the fastest growth rate among all categories, with an average annual growth rate of 12.54 %. Conversely, the cumulative export value of Rifles and other sports shooting equipment was only $0.213 billion, accounting for less than 1 % of the overall export value on average. This underscores the relatively underdeveloped state of China's sports shooting product sector in comparison to other sporting goods.

China's exports of sporting goods to non-Belt and Road countries and regions exhibit noteworthy diversification across a spectrum of products. Within this export framework, China not only maintains a resilient export performance in key products such as sports footwear, gymnastics and track and field equipment, and ball sports equipment and supplies, but also exhibits adaptability by making nuanced adjustments in response to market competition and evolving demands in diverse regional contexts. This diversified export structure attests to the strategic adaptability of Chinese sporting goods enterprises in the global market, enabling them to effectively navigate intense international market competition and cater to varied consumer preferences in different countries. Ultimately, this dynamic export approach creates a more conducive environment for the sustainable development of Chinese sporting goods in non-Belt and Road countries and regions.

Regarding exports to countries and regions along the “Belt and Road”, China's exports are predominantly dominated by gymnastics and athletics equipment and supplies, with a cumulative export value of $7.575 billion in Fig. 4, and the average annual export value is $0.583 billion. Sports footwear constitute the second-highest category in terms of exports. Notably, the fastest-growing categories in China's exports to the “Belt and Road” countries and regions are sports fields and related equipment, and water sports equipment and supplies, with average annual growth rates of 14.30 % and 12.27 %, respectively. Conversely, the development level of rifles and other sports shooting equipment, and ice and snow sports equipment and supplies, appears to be insufficient. The average annual export value of rifles and other sports shooting equipment is $0.001 billion, while the average annual export value of sportswear and protective gear is $0.006 billion.

As the “Belt and Road” initiative advances, China's export of sporting goods to “Belt and Road” countries and regions encounters novel opportunities and challenges. Driven by policy initiatives, China intensifies sports cooperation with countries and regions along the “Belt and Road”, facilitating the bidirectional circulation of relevant products. Adjustments in the export product structure are now finely tailored to local cultures and consumer demands, meticulously considering the distinctive requirements of each country's or region's sports development. This context-specific export strategy not only enhances the market share of Chinese sporting goods in “Belt and Road” countries and regions but also establishes a robust foundation for mutually beneficial cooperation in the sports industry between the involved parties.

In conclusion, China exports a wide variety of sporting goods categories, indicating a comprehensive product range. However, there is a significant imbalance in the product structure, which is manifested in a polarized nature of exports. The developmental features of China's sporting goods export structure are influenced by a multitude of factors. Firstly, the export composition of sporting goods is subject to the impact of international market demand and trends. Certain products may dominate the export landscape due to their heightened demand in the global market, while others may face constraints arising from relatively low or fluctuating demand. Secondly, China's elevated manufacturing capabilities in specific sporting goods sectors propel the export of related products. Domestic industrial policies and investments emerge as pivotal factors in shaping the export structure of sporting goods, with the government actively endorsing specific industries or fostering technological innovations to stimulate exports. The influence of international sports events and activities, coupled with the impetus from trade collaborations and the “Belt and Road” initiative, further exerts an impact on the export structure of sporting goods [40,41]. Additionally, institutional elements, industry competition, and corporate strategies all contribute to molding the product structure of China's sporting goods exports. Consequently, to foster a more diversified and sustainable expansion of sporting goods exports, future Chinese policies and strategies should holistically consider these factors. By pursuing a more equitable trajectory of product development, enhancements in the export product structure of sporting goods can be achieved, facilitating a more diversified and sustainable array of sporting goods exports [42].

### 3.3 Export country characteristics

**Fig. 5:**
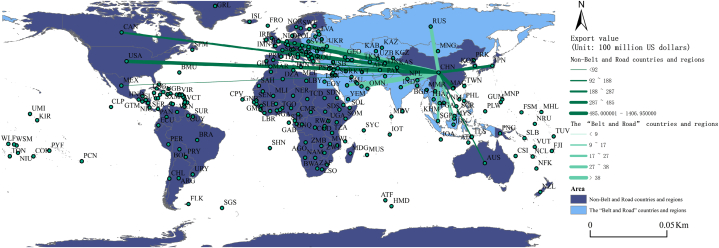
The cumulative total exports of sporting goods from China to the “Belt and Road” and non-Belt and Road countries and regions from 2007 to 2019, ranked in the top fifteen (Unit: 100 million US dollars).

Table 5 presents the cumulative export value rankings of China's sporting goods to the top 15 countries and regions along the “Belt and Road” and non-Belt and Road from 2007 to 2019. Fig. 5 is a visual map. Notably, there exists a significant difference in economic development levels and development vitality between non-Belt and Road countries and regions, which are predominantly developed countries, and the “Belt and Road” countries and regions, which consist largely of developing nations. As depicted in Table 5, among non- “Belt and Road” countries and regions, the United States emerges as the leading destination for China's sporting goods exports, with a substantial cumulative export value of $140.695 billion, nearly three times higher than the second-ranked Hong Kong. As shown in Fig. 5, the top 10 countries and regions in terms of export value are primarily located in North America, Western Europe, East Asia, and Australia, reflecting the prominence of these regions in China's sporting goods trade. In contrast, among the “Belt and Road” countries and regions, Russia stands out as the primary recipient of China's sporting goods exports, with a cumulative export value of $6.219 billion, ranking 14th overall. The top 15 countries and regions in terms of export value are predominantly concentrated in Eastern Europe, Western Asia, Southeast Asia, and South Asia, underscoring the significance of these regions as emerging markets for China's sporting goods.

**Table 5:** The cumulative total exports of sporting goods from China to the “Belt and Road” and non-Belt and Road countries and regions from 2007 to 2019, ranked in the top fifteen (Unit: 100 million US dollars).

Rank	The “Belt and Road” countries and regions		Non-Belt and Road countries and regions	
	Country name	Total exports	Country name	Total exports
1	Russian Federation	62.19	United States of America	1406.95
2	Poland	57.51	Hong Kong	485.22
3	United Arab Emirates	38.72	Japan	456.02
4	Saudi Arabia	27.44	Germany	397.30
5	Singapore	27.37	Great Britain	287.76
6	Czech Republic	25.44	France	223.97
7	India	23.48	Canada	188.22
8	Malaysia	22.01	Netherlands	142.54
9	Thailand	17.66	Australia	133.06
10	Turkey	16.45	Spain	92.07
11	Indonesia	12.88	Mexico	77.27
12	Iran	9.01	South Korea	77.11
13	Vietnam	8.99	Belgium	66.39
14	Slovakia	8.28	Italy	48.76
15	The Philippines	8.12	Spain	92.06

The configuration of disparities in the export characteristics of Chinese sporting goods between “Belt and Road” countries and regions and non-Belt and Road countries and regions is shaped by a myriad of intricate and interconnected factors. Influential elements encompass geographical distribution, economic development levels, cultural backgrounds, trade policies, “Belt and Road” initiative cooperation, government support, and investments, as well as regional sports activities. These factors collectively contribute to shaping the demand and market preferences for sporting goods in distinct countries. China's role in advancing the “Belt and Road” initiative, coupled with its positive economic contributions to participating nations, significantly influences the dynamics of sporting goods trade. Furthermore, specific regional sports activities' prevalence, policy divergences, and uneven development in international trade cooperation are pivotal in determining the characteristics of export destinations. This multifaceted interplay results in diverse features of Chinese sporting goods exports across different countries, emphasizing the multi-layered and multidimensional nature of the global sports trade environment. Concurrently, within the framework of the international division of labor and global industrial shifts, China's sporting industry exhibits increasing economic vitality. This transformative process involves adopting innovative development models, fostering research and development capabilities, strengthening global economic connections, and enhancing international competitiveness and standing. These concerted efforts gradually elevate China's position in global sports goods trade, cultivating a diversified network of trade partners and assuming an increasingly pivotal role in shaping dynamics within the global sports industry. The data presented in Table 5 underscores the evolutionary nature of China's sporting goods exports, reflecting China's adaptability and strategic positioning amidst the evolving dynamics of the global economy. With the ongoing development and innovation in China's sports industry, there is the prospect of exerting a more significant influence on international sports goods trade, contributing to the country's economic growth, and promoting international cooperation and development in the field of sports.

### 3.4 Export network characteristics

**Fig. 6:**
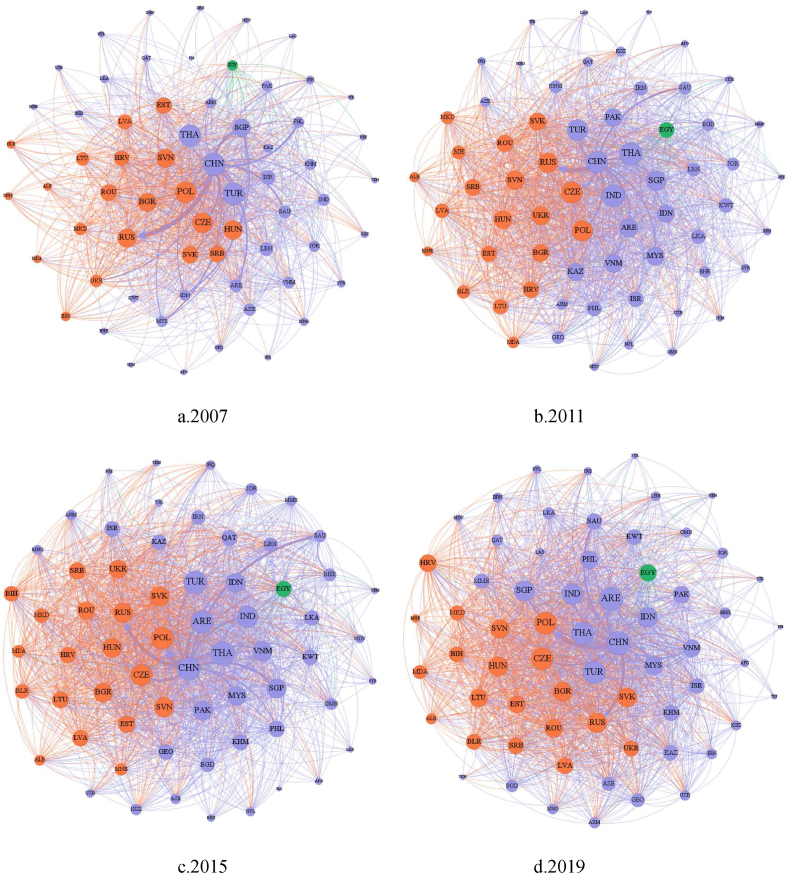
Visualization of the trade network of sporting goods along the “Belt and Road” initiative.

This paper conducts an in-depth analysis of the relationship between China and the countries and regions along the “Belt and Road” using social network analysis. It visually depicts the “Belt and Road” trade network for the years 2007, 2011, 2015 and 2019. The results are visually presented in Fig. 6. In this figure, nodes represent different countries and regions, and their size corresponds to the centrality of each country or region in the trade network, indicating their core relationships and influence. Larger nodes indicate a more central position with greater influence. The thickness of the edges between nodes represents the degree of trade in sporting products between different economies, with thicker edges indicating higher levels of trade in sporting goods between countries.

From Fig. 6, it becomes evident that the connections between countries and regions along the “Belt and Road” have grown increasingly frequent and robust over time, with trade volume steadily increasing each year. The “Belt and Road” trade network exhibits an “edge-core structure”, with China, as the initiator of the “Belt and Road” initiative, holding a prominent central position in the trade network. In 2007, in Fig. 6(a), Turkey, Thailand, China, Poland, the Czech Republic, Hungary, and the Russian Federation occupied central positions in the trade network. In Fig. 6(b), by 2011, Thailand emerged as the central node in the “Belt and Road” trade network, and countries like India, Singapore, and Slovenia gradually moved from relatively peripheral positions to more central ones, displacing countries such as Hungary in the trade network. In 2015, Thailand, China, Turkey, Poland, and the Czech Republic maintained their central positions, while the Russian Federation and Singapore shifted relatively towards the periphery in Fig. 6(c). By 2019, the United Arab Emirates further moved closer to the center, almost surpassing Thailand and Turkey in Fig. 6(d).

In summary, the analysis reveals that China holds a relatively central position in the trade dependency network of sporting goods along the “Belt and Road” initiative, underscoring its crucial role in facilitating and driving this trade network. The implementation of the “Belt and Road” Initiative, coupled with the economic development disparities among the countries and regions along the “Belt and Road”, has substantially heightened the economic dynamism and trade engagements within these regions. Consequently, this has fostered the establishment of the trade network. The dynamics of this trade network manifest a distinctive “edge-core structure”, with China positioned centrally. Nations such as Thailand, Turkey, Poland, the Czech Republic, Russia, along with emerging economies like Singapore and the United Arab Emirates, signify an escalating economic importance within the trade network. This underscores their growing economic influence in the Belt and Road sporting goods trade network. This comprehensive analysis reveals the intricacies of China's sporting goods trade network with countries and regions along the “Belt and Road”, emphasizing the collaborative efforts among nations in this domain and highlighting China's pivotal role in the global sports trade landscape.

## 4 The “Belt and Road” initiative and China's sporting goods exports: policy evaluation

### 4.1 Benchmark results

According to equation (1), this paper employs a fixed-effects model to examine the impact of the “Belt and Road” initiative on the export of sporting goods through the DID method. The regression results are presented in Table 6.

**Table 6:** Basic regression.

Explanatory variables	Interpreted variables: ln*export*			
	(1)	(2)	(3)	(4)
*post* × *treat*	0.216***	0.206***	0.179***	0.146**
	(0.057)	(0.059)	(0.061)	(0.063)
ln*pgdp*			0.950***	1.000***
			(0.177)	(0.182)
ln*oer*			−0.054	−0.069
			(0.063)	(0.065)
*openness*			0.010***	0.011***
			(0.004)	(0.004)
ln*pop*			2.545***	2.358***
			(0.304)	(0.314)
ln*dis*			1.717*	1.623
			(1.024)	(1.043)
*wto*			−0.181	−0.192
			(0.847)	(0.857)
*sport*			−0.279	−0.220
			(0.566)	(0.582)
_cons	6.113***	6.348***	−24.415***	−23.469***
	(0.188)	(0.515)	(8.519)	(8.690)
*N*	2301	2301	1730	1730
Year fixed effect	Yes	Yes	Yes	Yes
Country fixed effect	Yes	Yes	Yes	Yes
Category-year fixed effect	No	Yes	No	Yes

Note: ***, **, * indicate statistical significance at the 1 %, 5 %, and 10 % levels respectively. Robustness standard errors are reported in parentheses. Same as below.

The findings in column (1) reveal that the estimated coefficient of the interaction term *post* × *treat* is 0.216 and statistically significant at the 1 % level, after controlling for both year and country fixed effects. This suggests that, on average, the implementation of the “Belt and Road” initiative has had a positive and significant effect on China's sporting goods exports. Subsequently, in column (2), this paper incorporates the development characteristics of each sporting goods category in each year as fixed effects. Despite this adjustment, the estimated coefficient of *post* × *treat* is reduced to 0.206, but it remains statistically significant at the 1 % level. This further reinforces the notion that the “Belt and Road” initiative has exerted a positive impact on China's sporting goods exports. Columns (3) and (4) further explore the policy effects of the “Belt and Road” initiative by introducing additional control variables with two-way fixed effects for year and country, and without controlling for category-year effects, respectively. The presence of missing values in the control variables leads to some variation in the sample of estimates in these two columns compared to the previous ones. Nonetheless, the results continue to indicate a positive impact of the “Belt and Road” initiative on China's sporting goods exports. Specifically, after controlling for year, country, and category, the estimated coefficient of *post* × *treat* is 0.146, which is statistically significant at the 5 % level. This implies that the implementation of the “Belt and Road” initiative has had a catalytic effect on China's sporting goods exports, even when considering other potential explanatory factors and controlling for various fixed effects.

The results from column (4) of the analysis reveal several notable findings regarding the impact of the control variables on China's sporting goods exports. The variable ln*pgdp* exhibits a significant positive effect on ln*export*, suggesting that countries with higher levels of economic development tend to import more sporting goods from China. This observation aligns with economic intuition, as countries with stronger economies are likely to have greater purchasing power and demand for consumer goods like sporting goods. The variable ln*oer* shows a negative estimation coefficient and fails the significance test. This indicates that greater fluctuations in exchange rates may lead to economic instability in a country, which in turn may negatively impact its sporting goods trade. The variable *openness* displays a significantly positive coefficient at the 1 percent level. It can be inferred that countries with more open economies are likely to receive more foreign investment and consequently support the export of sporting goods from China to these countries. The variable ln*pop* demonstrates a significant positive impact on sporting goods exports and is significant at the 1 percent level. This means that countries with a higher population density tend to have larger markets and therefore a higher demand for sporting goods, which leads to an increase in exports from China. The variable ln*dis* shows a positive estimation coefficient but fails the significance test. The variables *wto* and *sport* both display negative estimation coefficients and do not pass the significance test.

### 4.2 Dynamic effects testing

**Fig. 7:**
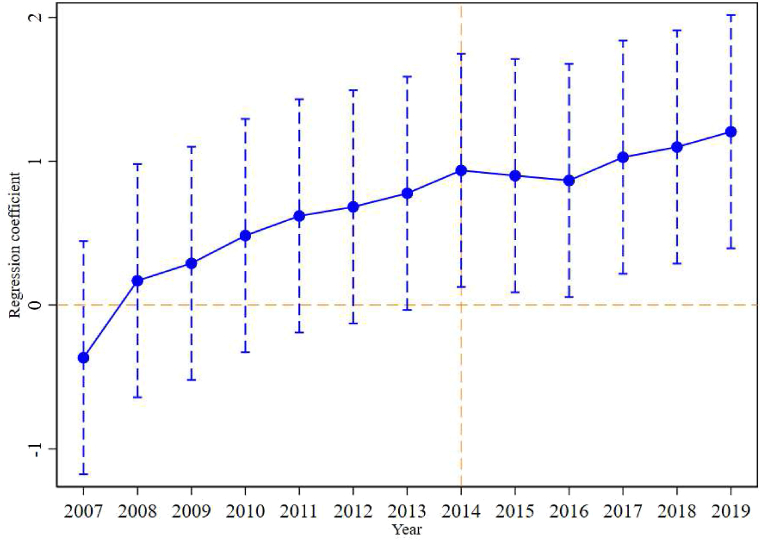
Dynamic effects of the “Belt and Road” initiative on the Export Value of Sporting Goods.

Furthermore, Fig. 7 illustrates the dynamic effect of the “Belt and Road” initiative on the export value of sporting goods. The results indicate that the coefficient of the interaction term *post* × *treat* is not statistically significant for the first seven years following the policy implementation, providing evidence in support of the parallel trend hypothesis in the DID estimation. This finding suggests that prior to the implementation of the “Belt and Road” initiative, the trends in sporting goods exports for the treatment group (countries and regions along the “Belt and Road”) and the control group (other countries and regions) were similar and comparable. After the implementation of the “Belt and Road” initiative in 2014, the export value of sporting goods exhibited a significant and sustained increase for six consecutive years. This finding implies that the “Belt and Road” initiative has had a substantial positive impact on China's sporting goods exports to countries and regions along the “Belt and Road”. The notable growth in exports indicates that the initiative has been effective in promoting and enhancing trade relationships in the sporting goods sector between China and the “Belt and Road” countries and regions.

**Fig. 8:**
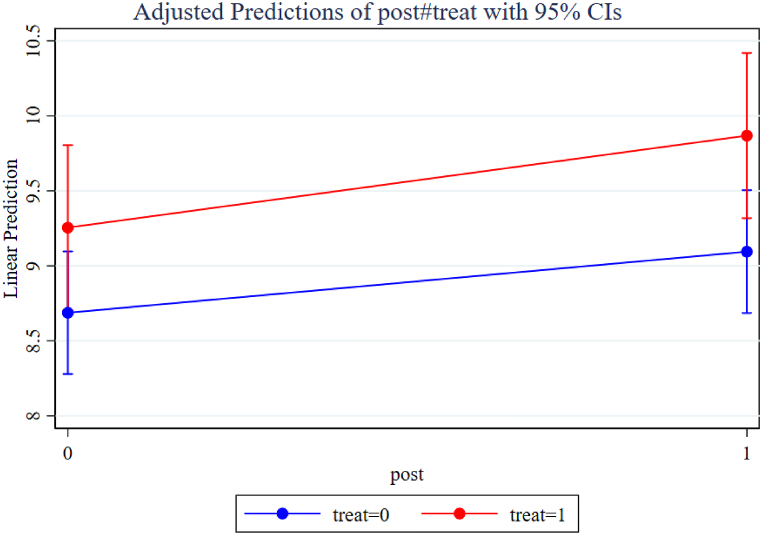
Marginal effects of the “Belt and Road” initiative on the Export Value of Sporting Goods.

To analyze the marginal effects of the “Belt and Road” initiative on sporting goods exports, this paper computes the predicted marginal value of policy implementation with a 95 percent confidence interval (Table 7). Fig. 8 presents the marginal effects of the “Belt and Road” initiative on sporting goods exports. The results demonstrate that both before and after the implementation of the policy, there exists a notable difference between the forecasted marginal values of non-Belt and Road countries and regions, which amounts to 0.238. In comparison, the difference between the forecasted marginal values of countries and regions along the “Belt and Road” is larger, reaching 0.385. This disparity in marginal impacts indicates that the implementation of the policy exerts a more substantial effect on countries and regions along the “Belt and Road”, resulting in a net effect of 0.146. The findings suggest that the “Belt and Road” initiative has a statistically significant and positive marginal effect on China's sporting goods exports, particularly for the countries and regions participating in the initiative. The calculated net effect of 0.146 signifies an overall increase in sporting goods exports, highlighting the initiative's role in fostering trade cooperation and economic growth in the sporting goods sector with the “Belt and Road” partner countries and regions.

**Table 7:** Marginal effects of the “Belt and Road” initiative on the Export Value of Sporting Goods.

		Marginal effects	Single differential	DID
Control group treat = 0	Before the policy	8.694***	0.238*** (0.044)	0.146** (0.063)
	post = 0	(0.207)		
	After the policy	8.932***		
	post = 1	(0.207)		
Treatment groups treat = 1	Before the policy	9.376***	0.385*** (0.059)	
	post = 0	(0.296)		
	After the policy	9.762***		
	post = 1	(0.298)

### 4.3 Robustness test

In consideration of the substantial sample size used in this paper for calculating sporting goods exports and to address potential concerns regarding outliers' influence on research findings, the dependent variable ln*export* was winsorized (*wlnexport*). To ensure the accuracy and robustness of the results presented in this paper, we used this data transformation approach. The regression results are reported in Table 8. Notably, even after winsorization was applied to the dependent variable ln*export*, the estimated coefficient of the interaction term *post × treat* on the *wlnexport* remained significantly positive. Additionally, there was minimal change observed in the coefficient compared to the baseline regression results, even after controlling for fixed year, country, and category-year effects. This indicates that the positive impact of the “Belt and Road” initiative on China's sporting goods exports persisted and remained statistically significant. The consistency of the coefficient's significance and stability in the regression analysis further strengthens the evidence supporting the conclusion that the “Belt and Road” initiative effectively stimulated China's export of sporting goods.

**Table 8:** Robustness test.

Explanatory variables	Interpreted variables: *wlnexport*		
	(1)	(2)	(3)
*post* × *treat*	0.223***	0.185***	0.149**
	(0.054)	(0.057)	(0.059)
_cons	6.152***	−23.537***	−22.985***
	(0.177)	(8.065)	(8.233)
*N*	2301	1730	1730
Control variables	No	Yes	Yes
Year fixed effect	Yes	Yes	Yes
Country fixed effect	Yes	Yes	Yes
Category-year fixed effect	No	No	Yes

### 4.4 Mechanism analysis

Based on the previous analysis, it becomes evident that the implementation of the “Belt and Road” initiative has a positive impact on promoting the export of Chinese sporting goods. However, the specific effect of the “Belt and Road” initiative on the growth of sporting goods exports remains uncertain. To examine the mechanism of policy implementation on sporting goods exports in more detail, this paper categorizes the growth of sporting goods exports into two dimensions: extensive margin and intensive margin. The results of this analysis are presented in Table 9. As observed from Table 9, the effect of *post* × *treat* on the extensive margin, while controlling for other factors, is found to be negative and statistically significant at the 5 % level. This indicates that the implementation of the policy may inhibit the growth of sporting goods exports in terms of product breadth. The “Belt and Road” initiative aims to strengthen economic ties between China and the countries and regions along the route, promoting trade and investment cooperation. As a result, Chinese sports goods exporters can more easily export their products to these countries and regions. However, cultural, linguistic, and market environmental differences in these areas may pose challenges to Chinese sports goods exporters. To reduce the difficulty and cost of entering the market, Chinese sports goods exporters may prefer to concentrate their export markets in these areas, resulting in the phenomenon of regional concentration of export destinations. Thus, the trend of narrowing the scope of sports goods exports and increasing the concentration of export destinations is consistent with the regional concentration trend identified in the previous analysis. Additionally, Chinese sports goods exporters may prefer to export their products to these countries and regions due to the fact that the market demand in these areas is more suitable for their products. For example, some countries and regions place greater emphasis on outdoor sports, and Chinese sports goods exporters may be more willing to export outdoor sports products to these countries and regions to meet market demand. This may also contribute to the concentration of export destinations. In contrast, the effect of *post* × *treat* on the intensive margin, considered as a whole, and on the price margin is positive but does not achieve statistical significance. However, the effect of *post* × *treat* on the quantity margin is significantly positive at the 10 % level. This suggests that the implementation of the “Belt and Road” policy has a positive contribution to sporting goods exports in terms of product depth, primarily driven by an increase in export quantity. This may be because the market demand in some countries and regions along the route places greater emphasis on price and quantity rather than quality and added value. To meet the demands of these markets, Chinese sports goods exporters may focus more on exporting greater quantities of products to these countries and regions, rather than higher quality products. This may result in the intensive margin of growth in Chinese sports goods exports being primarily driven by the quantity margin. As a result, Chinese sports goods exporters may prioritize quantity growth over quality and added value improvements when exporting products to countries and regions along the route. In addition, Chinese sports goods exporters may face other challenges such as market access barriers and intellectual property protection issues, which may limit their ability to improve product quality and added value. Therefore, they may prioritize quantity growth to meet market demand and maintain competitiveness. Therefore, there is a call for continued efforts to enhance the quality of sporting goods exports. By optimizing and upgrading the product mix and focusing on product improvement, China can fully capitalize on the opportunities presented by the “Belt and Road” initiative to elevate the competitiveness and global standing of its sporting goods industry.

**Table 9:** Mechanism analysis.

Explanatory variables	Extensive margin EM	Intensive margin		
		IM	P	Q
	(1)	(2)	(3)	(4)
*post* × *treat*	−0.0341**	0.0175	0.0174	0.0005*
	(0.0153)	(0.0120)	(0.0120)	(0.0002)
_cons	−8.3311***	−10.6793***	−10.6755***	0.9285***
	(2.1284)	(1.6641)	(1.6658)	(0.0339)
*N*	1730	1730	1730	1730
Control variables	Yes	Yes	Yes	Yes
Year fixed effect	Yes	Yes	Yes	Yes
Country fixed effect	Yes	Yes	Yes	Yes
Category-year fixed effect	Yes	Yes	Yes	Yes

## 5 Discussion

Based on the current literature review, it is evident that there is a dearth of research focusing on the evaluation of policy effectiveness within the “Belt and Road” initiative, despite the considerable international attention it has attracted. This study distinguishes itself from prior research in the sports industry by emphasizing the assessment of policy effects, whereas earlier work has predominantly concentrated on the countries and regions along the “Belt and Road”. This distinction may lead to disparities in the research methods required and employed. This paper adopts the DID method to ascertain whether the implementation of the “Belt and Road” initiative has contributed to China's sporting goods exports. This methodological choice represents a significant advancement in the domain of policy evaluation methods within sports industry research. The use of DID estimation is well-established globally for evaluating the efficacy of public policies [43,44]. In this context, the DID method is employed to discern the impact of the “Belt and Road” initiative's implementation on China's sporting goods exports, thereby capturing the policy effects between the treatment and control groups. The theoretical contribution of this paper lies in applying the DID method to estimate the policy effects of the “Belt and Road” initiative on sporting goods exports. Empirical data on China's sporting goods exports from 2007 to 2019 illustrates that the implementation of the “Belt and Road” initiative has yielded an overall direct and statistically significant positive effect on China's sporting goods exports. Through DID method estimation, a comprehensive understanding of the binary marginal effect of the “Belt and Road” initiative on China's sports goods exports is obtained, enabling the evaluation of the initiative's impact on export growth, destination concentration, and the marginal contributions of these changes to export volumes. Rigorous tests and robustness checks of the DID method validate the significance and resilience of the results. Furthermore, in contrast to the work of Ji and Ren [6], Liang and Wang [11], Wu et al. [23], Chen and Liu [24], this study's feature analysis integrates social network analysis with traditional quantitative analysis, thereby constructing the “Belt and Road” trade network to investigate distribution patterns and competitive dynamics within the trade network. The social network analysis method provides us with a global perspective for a deeper understanding of the trade network structure under the “Belt and Road” initiative. The trade network of the “Belt and Road” initiative involves various participating entities from multiple countries and regions, including governments, businesses, and non-governmental organizations, making it more diverse compared to traditional trade network. Its forms of cooperation are more diversified, encompassing not only traditional goods trade but also service trade, investment cooperation, and other forms, providing more options for trade cooperation among countries. Through the social network analysis method, we can gain insights into the trade relationships among different countries and regions, including the flow of sports goods trade, trade partner relationships, and key nodes within the trade network. This helps in identifying strategic sports goods trade partners under the “Belt and Road” initiative, providing strategic guidance for China's development as a sports power. Furthermore, social network analysis enables us to understand the strength of trade relationships between different countries and regions, i.e., their level of cooperation in sports goods trade. The various nodes in the “Belt and Road” trade network are interconnected, forming a complex network structure that facilitates information sharing, resource integration, and provides guidance for future cooperation. In addition, social network analysis also helps in understanding the competitive situation within the sports goods trade network under the “Belt and Road” initiative. By analyzing the trade network structure, we can identify competitors, market shares, and competitive advantages, providing guidance for the formulation of trade policies and cooperation agreements. The “Belt and Road” trade network promotes economic regional integration among participating countries and regions, strengthening economic ties and cooperation, and driving the overall economic development of the region. Simultaneously, the “Belt and Road” trade network fosters exchange and cooperation among different cultures, promoting cultural diversity and global development. Through the analysis of trade network structure, we can understand China's position within the sporting goods trade network, providing meaningful guidance for the formulation of trade policies and cooperation agreements. In conclusion, the “Belt and Road” trade network, within the context of building a sports power, exhibits characteristics of increased diversification, network development, regionalization, and cross-cultural exchange, offering new opportunities and challenges for trade cooperation and economic development among countries.

## 6 Concluding remarks

### 6.1 Conclusion

In this paper, situated within the strategic context of China's sporting power, a systematic analysis of the fundamental characteristics of China's sporting goods exports to countries and regions along the “Belt and Road” is conducted. Moreover, the policy effect of the “Belt and Road” initiative on China's sporting goods exports is evaluated using the DID method. The main conclusions are as follows: Firstly, from 2007 to 2019, the cumulative export value of sporting goods to countries and regions exhibited a modest increase from $37.79 billion to $37.86 billion. The growth rate of sporting goods exports demonstrated fluctuations, but the overall trend displayed a stable upward trajectory. The export of sporting goods encompasses a diversified range of products, yet it is predominantly concentrated in categories such as gymnastics and track and field equipment, sports footwear, and ball sports equipment and supplies. On the other hand, the development level of certain categories, such as Rifles and other sports shooting equipment, remains relatively inadequate. Regarding the geographic distribution, the export areas of sporting goods span a wide range, but the distribution of export destinations exhibits significant variation, showing a trend of concentration. Southeast Asia, West Asia, and Eastern Europe emerge as the principal regions with concentrated sporting goods exports. Secondly, countries engaging in sporting goods trade similarly display development characteristics, with the trade concentration primarily observed in countries with higher levels of economic development, such as Thailand, Turkey, Russia, Poland, the United Arab Emirates, and Singapore. Thirdly, The results of the policy evaluation affirm that the implementation of the “Belt and Road” initiative has a positive impact on China's sporting goods exports. However, it is essential to note that the policy implementation is more conducive to the intensive margin of sporting goods exports rather than the extensive margin. This impact is predominantly reflected in the quantity of export products, rather than the quality of export products.

### 6.2 Suggestions

First, it is imperative to fully leverage the “Belt and Road” initiative to expand foreign markets and counter the monopoly of Western countries. In the context of increasing anti-globalization sentiments, there has been a surge in international trade frictions and resistance, leading to heightened trade uncertainty. Additionally, the COVID-19 pandemic has significantly disrupted global value chains, contributing to a widespread economic downturn. In this backdrop, the “Belt and Road” initiative presents an opportunity to effectively reduce trade costs, mitigate trade risks, and accelerate the overall upgrading of China's sporting industry within the global value chain [45]. By enhancing China's international influence and competitiveness, the initiative plays a pivotal role in promoting the export of Chinese sporting goods [17]. At present, the “Belt and Road” initiative has notably facilitated the outward extension of China's sporting goods exports, resulting in increased overall export volume and breadth. To capitalize on this potential, several strategic measures can be adopted. Firstly, engaging in trade investment and social marketing and establishing overseas offices in countries and regions along the “Belt and Road” can help navigate trade barriers, reduce investment constraints, and expand market reach through targeted promotion, effectively exploring new international markets. Investing to gain control over trade channels and participating in international competition can further expand the market share of sporting goods exports and reduce overreliance on a few export destinations. This approach can also drive specialization and segmentation in the international division of labor within the sporting industry, optimizing resource allocation and compensating for any inadequacies in production factors. Secondly, fostering cooperation in infrastructure projects, such as the development of “China-Europe freight trains” in ports, railways, and roads, is crucial. Strengthening national infrastructure and improving trade transportation channels can effectively reduce trade costs, including transaction and transportation costs. This, in turn, enhances production efficiency, fosters increased trade exchanges among countries, and facilitates interconnection within the sporting goods industry, ultimately leading to economies of scale.

Second, China must steadfastly adhere to the fundamental national policy of reform and opening-up while implementing the development principle of steady progress and promoting supply-side structural reforms and high-quality development in the sporting industry. China should actively promote the construction of a new pattern of comprehensive opening-up, seeking to achieve an organic unity between opening to the outside world and internal market openness. This involves establishing a unified domestic market and harnessing the advantages of its large economy. By opening up the market, China can mitigate the impact of uncertainties in the international market. Simultaneously, the country must establish sound and comprehensive sports-related laws and regulations, facilitating the modernization of sports governance systems and capabilities. The establishment of dedicated sports management and enforcement departments is essential to ensure that the concept of the rule of law is effectively implemented in practical work, ensuring adherence to commitments in the sporting industry.

Third, the development of China's sporting industry has been primarily focused on manufacturing, while the growth of the sporting service industry has been comparatively slower. The 2021 Tokyo Olympics showcased the outstanding performance of the Chinese team, ranking second in both the number of gold medals and total medals. This success is attributed to the dedication and training of athletes, as well as the support provided by the country for sports development. Furthermore, numerous sports brands have demonstrated an integration of technology into the design of athletes' competition attire, contributing to their achievements in various competitions. In light of the opportunities presented by the information age, China should strategically leverage the development of information technology, including big data, artificial intelligence, and internet technology, to drive innovation in the sporting goods industry. The integration of these technological capabilities can empower the development of high-end manufacturing for sporting goods and optimize the trade structure of sporting products. Emphasizing research and development and the application of cutting-edge technologies such as 5G and virtual reality (VR) is essential in enhancing the sports and fitness sector to meet the current consumers' demands for immersive visual experiences in sporting events and home fitness. Moreover, by effectively integrating sporting events with other industries, China can effectively produce sports resources and promote the growth of sporting services, including sporting marketing, event operations, and sporting tourism. This holistic approach to sporting industry development will facilitate an upgrade in the industry's structure and significantly enhance the international competitiveness of sporting goods.

### 6.3 Research shortcomings and outlook

Certainly, there are aspects of this paper that still need improvement. The research of this paper focuses on national-level sporting goods exports due to data availability, which limits the reflection of more detailed phenomena at the city or enterprise levels, as seen in other studies. This limitation arises from the extensive period of the paper. Despite adopting a unified standard for defining sporting goods, this paper may still face data inaccuracies due to inconsistencies between international and domestic data sources, variations in statistical methods, and standards among different databases. Finally, considering the limitations of the data, there exist discrepancies in the samples used for the feature analysis and empirical analysis sections of this paper, with some countries' data missing. This might moderately affect the comprehensiveness of the results presented in this article. In addition, this paper will consider more external factors, such as the COVID-19 pandemic and the China-U.S. trade war in future research, and investigate data related to China's sporting goods trade agreements, hosting of international sporting events and sporting event participation to make the findings more rigorous. The objective of these investigations is to clarify the benefits of the “Belt and Road” initiative and analyze ways to enhance the progress of sporting goods development while ensuring its stability.

## Data availability statement

Data included in article/supp, material/referenced in article.

## Funding

The study was supported by Social Science Foundation of Guangdong Province (GD19CTY07) and the 14th Five Year Plan for the Development of Philosophy and Social Sciences in Guangzhou City (2022GZYB63).

## CRediT authorship contribution statement

**Rui Wang:** Writing – original draft, Software, Methodology, Data curation. **Tingting Liang:** Writing – review & editing, Funding acquisition, Formal analysis.

## Declaration of competing interest

The authors declare that they have no known competing financial interests or personal relationships that could have appeared to influence the work reported in this paper.

## Figures and Tables

**Table:** 

Country name	ISO3	Country name	ISO3	Country name	ISO3	Country name	ISO3	Country name	ISO3
Afghanistan	AFG	Czech Republic	CZE	Israel	ISR	New Zealand	NZL	Suriname	SUR
Albania	ALB	Democratic Republic of the Congo	COD	Italy	ITA	Nicaragua	NIC	Swaziland	SWZ
Algeria	DZA	Denmark	DNK	Jamaica	JAM	Niger	NER	Sweden	SWE
Andorra	AND	Djibouti	DJI	Japan	JPN	Nigeria	NGA	Switzerland	CHE
Angola	AGO	Dominica	DMA	Jordan	JOR	North Korea	PRK	Syria	SYR
Argentina	ARG	Dominican Republic	DOM	Kazakhstan	KAZ	Norway	NOR	Tajikistan	TJK
Armenia	ARM	Ecuador	ECU	Kenya	KEN	Oman	OMN	Tanzania	TZA
Aruba	ABW	Egypt	EGY	Kiribati	KIR	Pakistan	PAK	Thailand	THA
Australia	AUS	El Salvador	SLV	Kuwait	KWT	Palestinian territories	PSE	The Bahamas	BHS
Austria	AUT	Equatorial Guinea	GNQ	Kyrgyzstan	KGZ	Panama	PAN	The Philippines	PHL
Azerbaijan	AZE	Estonia	EST	Laos	LAO	Papua New Guinea	PNG	Timor Leste	TLS
Bahrain	BHR	Ethiopia	ETH	Latvia	LVA	Paraguay	PRY	Togo	TGO
Bangladesh	BGD	Fiji	FJI	Lebanon	LBN	Peru	PER	Tonga	TON
Barbados	BRB	Finland	FIN	Liberia	LBR	Poland	POL	Trinidad & Tobago	TTO
Belarus	BLR	France	FRA	Libya	LBY	Portugal	PRT	Tunisia	TUN
Belgium	BEL	French polynesia	PYF	Lithuania	LTU	Qatar	QAT	Turkey	TUR
Belize	BLZ	Gabon	GAB	Luxembourg	LUX	Republic of Macedonia	MKD	Turkmenistan	TKM
Benin	BEN	Gambia	GMB	Macao	MAC	Republic of the Congo	COG	Uganda	UGA
Bolivia	BOL	Georgia	GEO	Madagascar	MDG	Romania	ROU	Ukraine	UKR
Bosnia & Herzegovina	BIH	Germany	DEU	Malawi	MWI	Russian Federation	RUS	United Arab Emirates	ARE
Botswana	BWA	Ghana	GHA	Malaysia	MYS	Rwanda	RWA	United States of America	USA
Brazil	BRA	Great Britain	GBR	Maldives	MDV	Samoa	WSM	Uruguay	URY
Brunei	BRN	Greece	GRC	Mali	MLI	Saudi Arabia	SAU	Uzbekistan	UZB
Bulgaria	BGR	Greenland	GRL	Malta	MLT	Senegal	SEN	Vanuatu	VUT
Burkina	BFA	Guatemala	GTM	Mauritania	MRT	Serbia	SRB	Venezuela	VEN
Cote d'Ivoire	CIV	Guinea	GIN	Mauritius	MUS	Seychelles	SYC	Vietnam	VNM
Cambodia	KHM	Guyana	GUY	Mexico	MEX	Sierra Leone	SLE	Yemen	YEM
Cameroon	CMR	Haiti	HTI	Moldova	MDA	Singapore	SGP	Zambia	ZMB
Canada	CAN	Honduras	HND	Mongolia	MNG	Slovakia	SVK	Zimbabwe	ZWE
Cape Verde	CPV	Hong Kong	HKG	Montenegro	MNE	Slovenia	SVN		
Central African Republic	CAF	Hungary	HUN	Morocco	MAR	Solomon Islands	SLB		
Chile	CHL	Iceland	ISL	Mozambique	MOZ	Somalia	SOM		
Colombia	COL	India	IND	Myanmar	MMR	South Africa	ZAF		
Costa Rica	CRI	Indonesia	IDN	Namibia	NAM	South Korea	KOR		
Croatia	HRV	Iran	IRN	Nepal	NPL	Spain	ESP		
Cuba	CUB	Iraq	IRQ	Netherlands	NLD	Sri Lanka	LKA		
Cyprus	CYP	Ireland	IRL	New Caledonia	NCL	St. Lucia	LCA		
